# Health Education through Analogies: Preparation of a Community for Clinical Trials of a Vaccine against Hookworm in an Endemic Area of Brazil

**DOI:** 10.1371/journal.pntd.0000749

**Published:** 2010-07-20

**Authors:** Maria Flavia Gazzinelli, Lucas Lobato, Leonardo Matoso, Renato Avila, Rita de Cassia Marques, Ami Shah Brown, Rodrigo Correa-Oliveira, Jeffrey M. Bethony, David J. Diemert

**Affiliations:** 1 School of Nursing, Universidade Federal de Minas Gerais, Belo Horizonte, Minas Gerais, Brazil; 2 Albert B. Sabin Vaccine Institute, Washington, District of Columbia, United States of America; 3 Centro de Pesquisas René Rachou, Belo Horizonte, Minas Gerais, Brazil; 4 Department of Microbiology, Immunology and Tropical Medicine, The George Washington University, Washington, District of Columbia, United States of America; Federal University of Minas Gerais, Brazil

## Abstract

**Background:**

Obtaining informed consent for clinical trials is especially challenging when working in rural, resource-limited areas, where there are often high levels of illiteracy and lack of experience with clinical research. Such an area, a remote field site in the northeastern part of the state of Minas Gerais, Brazil, is currently being prepared for clinical trials of experimental hookworm vaccines. This study was conducted to assess whether special educational tools can be developed to increase the knowledge and comprehension of potential clinical trial participants and thereby enable them to make truly informed decisions to participate in such research.

**Methodology/Principal Findings:**

An informational video was produced to explain the work of the research team and the first planned hookworm vaccine trial, using a pedagogical method based on analogies. Seventy-two adults living in a rural community of Minas Gerais were administered a structured questionnaire that assessed their knowledge of hookworm, of research and of the planned hookworm vaccine trial, as well as their attitudes and perceptions about the researchers and participation in future vaccine trials. The questionnaire was administered before being shown the educational video and two months after and the results compared. After viewing the video, significant improvements in knowledge related to hookworm infection and its health impact were observed: using a composite score combining related questions for which correct answers were assigned a value of 1 and incorrect answers a value of 0, participants had a mean score of 0.76 post-video compared to 0.68 pre-video (*p* = 0.0001). Similar improvements were seen in understanding the purpose of vaccination and the possible adverse effects of an experimental vaccine. Although 100% of participants expressed a positive opinion of the researchers even before viewing the film and over 90% said that they would participate in a hookworm vaccine trial, an increase in the number who expressed fear of being vaccinated with a novel vaccine was seen after viewing the video (51.4% post-video versus 29.2% pre-video). Increases were also seen in the proportion who thought that participation in a vaccine trial would be inconvenient or disrupt their daily activities.

**Conclusions/Significance:**

Even in rural, resource-limited populations, educational tools can be specially designed that significantly improve understanding and therefore the likelihood of obtaining truly informed consent for participation in clinical research. The observed changes in the knowledge and perceptions of the research participants about hookworm infection and the experimental hookworm vaccine demonstrate that the video intervention was successful in increasing understanding and that the subjects acquired knowledge pertinent to the planned research.

## Author Summary

Conducting clinical trials of new vaccines in rural, resource-limited areas can be challenging since the people living in these areas often have high levels of illiteracy, little experience with clinical research, and limited access to routine health care. Especially difficult is obtaining informed consent for participation in this type of research and ensuring that potential participants adequately understand the potential risks and benefits of participation. The researchers have been preparing a remote field site in the northeastern part of the state of Minas Gerais, Brazil, for clinical trials of experimental hookworm vaccines. A special educational video was designed based on the method of analogies to introduce new scientific concepts related to the researchers' work and to improve knowledge of hookworm, a disease that is highly prevalent in their community. A questionnaire was administered both before and after the video was shown to a group of adults at the field site, which demonstrated the effectiveness of the video in disseminating knowledge about hookworm infection and about the vaccine being developed. Therefore, even in a rural, resource-limited area, educational tools can be specially designed that significantly improve understanding and therefore the likelihood of obtaining truly informed consent for participation in clinical research.

## Introduction

In research involving human subjects, the ethical relationship that must be established and maintained between investigators and research subjects is essential to successfully conduct investigational clinical trials of experimental drugs or vaccines, especially ones in which the participants are drawn from vulnerable populations [1], [2]. In such studies, investigators must attempt to mitigate any possible manipulation of the research population during the recruitment process, and especially to ensure that the risks and benefits to which volunteers are going to be exposed are well understood [3], [4]. The informed consent document is the traditional instrument utilized for this aim; by signing it, it is assumed that the volunteer has freely exercised their will, has formed their own evaluation and critique of the proposed research and has arrived at a truly informed decision about participation [5]–[8].

The process of obtaining informed consent becomes especially challenging when working in rural, resource-limited settings. Although there is no consensual definition of vulnerability, age, socioeconomic status, access to basic services such as health and sanitation, ethnic group, religion, cultural affiliation, and educational level are all characteristics that have been cited as indicating vulnerability, and which may therefore influence an individual's ability to consent to participation in clinical trials both in terms of the ability to exercise autonomy, but also to comprehend the proposed research [3], [9], [10]. With respect to the latter challenge, the informed consent form must frequently convey complex technical information and scientific concepts that are often not easily understandable, especially in populations with low levels of literacy [11]–[13]. To improve the quality of the information transmitted to potential study participants, researchers have, in general, increased the amount of information in informed consent documents [11]. However, a document that contains extensive and complex information may not convey a satisfactory understanding of the study procedures, or of the potential risks and benefits of participation in the research [14]. As an example, after evaluating the understanding of the information in an informed consent document for a research project in San Francisco, California, researchers found that despite using a form that had been simplified using language appropriate for a primary school level, the majority of individuals required more than one explanation of the study before satisfactorily comprehending it [12]. In that study, low literacy level and socioeconomic status were associated with an increased need for interventions that gave an improved comprehension of the information contained in the document.

In addition to the issue of comprehension, recruiting volunteers into clinical trials being conducted in resource-limited settings is further complicated by the limited access to medical care that is frequently found in such areas. Frequently, potential research subjects may feel an obligation to participate in order to receive medical attention for themselves or their family members [15]. Such motivation could influence individuals to participate and undertake risks that they otherwise would not accept.

Despite these very real issues related to obtaining informed consent from vulnerable populations living in resource-limited settings, it is often necessary to conduct clinical trials in such areas, particularly when the product being developed is meant to treat or prevent a disease that affects such a population. As one example, hookworm infection is one of the most prevalent chronic infections of humans, with an estimated 740 million cases worldwide, mostly in rural poor rural areas of the tropics and subtropics [16]. The two hookworms that infect humans are *Necator americanus* and *Ancylostoma duodenale*, with infection being transmitted through skin contact with soil contaminated with infective larvae. The major clinical manifestations result primarily from the loss of blood caused by adult worms that attach onto the intestinal wall, resulting in anemia which subsequently can lead to delays in cognitive development in children and reduction of well-being and productivity in adults [17].

Although effective chemotherapy exists to treat hookworm, current anthelminthics have important limitations, not least of which is that re-infection often occurs within a short time after treatment due to ongoing exposure [18], [19]. To develop an alternative control tool, the Human Hookworm Vaccine Initiative (HHVI) is developing a vaccine to prevent the morbidity due to this parasitic infection. Since hookworm does not occur in the developed world, testing the safety and efficacy of vaccines targeting this parasite must be conducted in the rural, resource-limited areas where the disease is endemic, among populations which are frequently referred to as being “vulnerable.”

The HHVI has been preparing a trial site for studies of its investigational hookworm vaccines that is based in the town of Americaninhas, in the northeast part of the Brazilian state of Minas Gerais [20], [21]. The first experimental vaccine being developed by the HHVI – the *Na*-ASP-2 Hookworm Vaccine – was tested there in a phase 1 clinical trial in 2007. For this study, healthy adult volunteers were recruited from communities surrounding Americaninhas.

In advance of this trial, studies were performed to assess the baseline knowledge of potential study participants in order to design appropriate educational interventions that could be used in the consenting process. Unfortunately, little is known about which are the more appropriate pedagogic models for informing populations involved in clinical trials, especially vulnerable populations that are economically or educationally disadvantaged. In the field of health education, purely informative pedagogic models do not usually result in modification of positions or attitudes, since behaviors are manifestations of firmly-held values and beliefs [22]. The ideal nature of an educational intervention that leads to acquisition of the knowledge necessary to make a conscious decision about participating in a research study is a matter of debate. Several authors have proposed a pedagogic model that makes use of the analogy [11], [23]. Analogies are useful tools for forming mental constructs that simplify or render familiar what an individual is attempting to understand [24]. The use of analogies can introduce new scientific concepts or alter previously held ideas [25], and can overcome barriers to learning by allowing an individual to make creative connections between pre-existing concepts and those related to the new knowledge being presented [23]. With this in mind, the current study aimed to develop an educational intervention based on analogies for a population resident in a hookworm endemic area of Brazil, and to evaluate its effectiveness in disseminating knowledge about the disease caused by hookworm, the experimental *Na*-ASP-2 vaccine that was about to be tested in their community, and about attitudes related to their willingness to participate in clinical trials of the vaccine.

## Methods

This research was conducted as part of a larger epidemiological study whose purpose was to establish the prevalence of hookworm infection in various communities in the study area, in advance of planned hookworm vaccine trials.

### Study Site

The study was conducted in 2007 in the communities of Furtado, Beija-Flor and Jamir, all of which are rural areas endemic for hookworm located in the region surrounding the town of Americaninhas in the municipality of Novo Oriente de Minas, 500 kilometers northeast of Belo Horizonte, the capital of the Brazilian state of Minas Gerais. Americaninhas is located in a mountainous region with a tropical climate [20], [26]. The population largely exists on subsistence farming of cassava, sugar cane, coffee and beans. They typically live in simple, hand-made dwellings of packed earth or adobe with roofs of corrugated iron.

The population of Americaninhas consists of around 1000 people living in the urban center, with another 4000 living in the surrounding rural areas in smaller hamlets. Approximately 500 people live in the communities of Furtado, Beija-Flor and Jamir. This region was chosen for conducting clinical trials of experimental hookworm vaccines in view of the elevated prevalences of helminth infections that have been found during previous studies performed by the research team in the area: 68% for the hookworm *N. americanus*, 45% for *Schistosoma mansoni*, and 49% for *Ascaris lumbricoides* [20]. Such high prevalences are the result of socioeconomic and environmental conditions that favor the transmission and development of hookworm, such as a warm and humid climate, a lack of basic sanitation, and the low socioeconomic status of the population.

### Study Population

Individuals were included in the study if they were between the ages of 16 and 50 years and had been resident in the study area for the previous 24 months; were infected with hookworm as determined during the course of the larger epidemiological study; and, had completed a course of treatment for hookworm with albendazole. All volunteers consented to participate in the research, as evidenced by their signature on the informed consent form approved by the ethical review committees of the Centro de Pesquisas Rene Rachou (part of the Fundação Oswaldo Cruz – FIOCRUZ) and the George Washington University Medical Center, and completion of a true/false questionnaire that assessed their understanding of the informed consent document. Volunteers had to respond correctly to all questionnaire questions prior to being considered for inclusion into the study. In the cases of volunteers unable to read, the consent form was read aloud to them in the presence of an independent witness who also signed the form after the volunteer affixed their thumbprint.

### Educational Intervention

The intervention was an educational video containing details about the proposed *Na*-ASP-2 vaccine trial, as well as explanations regarding the nature of research, the work of researchers, the reasons the area was chosen to test a vaccine against hookworm, and concepts of hookworm disease, vaccination, and the use of placebos in vaccine trials (Videos S1, S2, S3, S4, S5, S6, S7, S8, and S9). The video was filmed in the communities of Jamir and Beija Flor, and was produced using a pedagogical approach based on the use of analogies. In it, the daily experiences of local inhabitants, such as the cultivation of cassava, and the making of flour, sweets and cheese, are compared to the manufacturing of vaccines and to the experiments of researchers working in the laboratory. The characters featured in the film are actual inhabitants of the community who are representative of those from the rural interior of the state of Minas Gerais, thus facilitating identification of the viewer with the individuals on screen and enabling the learning process.

The film opens with scenes showing typical day in the town of Americaninhas, with people enjoying themselves in the town square or observing the main street from their windows (Video S1). After these initial images, the film transitions to describing the production of a traditional regional sweet. Each step in its production is shown, starting with cultivation of the sugar cane from which the basic ingredient is derived, followed by extraction of cane juice using a machine, preparation of the other ingredients, and combining these in specific quantities to create a final, high-quality product. Interspersed with these images are those of FIOCRUZ researchers working in the laboratory, using machines and instruments to assist them in discovering ideal components that, when combined in the correct amounts, may produce an effective vaccine. An analogy is thereby constructed between typical experiences of the region such as the production of sweets and the manufacturing of a vaccine against hookworm.

The video then shows people being interviewed about their knowledge of hookworm using language that is unique to the local population (Videos S2, S3, and S4). The individuals discuss the illness known locally as “amarelão” or the “illness of Jeca-tatu” (after a popular cartoon character from the early 20^th^ century), its mode of transmission, and the associated symptoms. Their perceptions of the researchers working in the area and what they're doing in the region, as well as the hookworm vaccine program and the possible adverse effects of such a vaccine, are also presented.

In the final part of the video, members of the HHVI team speak on camera about the hookworm vaccine project, to clarify details of the planned clinical trial (Videos S5, S6, S7, S8, and S9). The presenters explain why hookworm is endemic in their community, the criteria for inclusion in the forthcoming vaccine trial, the adverse effects that the vaccine might cause, as well as how the vaccine is made.

The video was shown to prospective vaccine trial participants in group sessions in their own communities. After presentation of the film, conversation was encouraged to discuss and debate what was viewed so that the presented knowledge could be consolidated and learned.

### Data Collection

Data were collected by means of a structured survey consisting of 45 questions, which was designed to assess knowledge about hookworm, vaccines (in general and the *Na*-ASP-2 vaccine in particular), and the researchers, as well as attitudes related to their willingness to participate in clinical trials of hookworm vaccines (Text S1). The questionnaire consisted of a combination of true or false questions, multiple choice questions, and subjective questions answered according to the 5-point Likert scale (ranging from “strongly disagree” to “strongly agree”). Survey questions were divided into three categories: a) those assessing knowledge about hookworm (Group 1); b) those assessing knowledge about the hookworm vaccine and upcoming clinical trial (Group 2); and, c) those assessing the attitudes and feelings of individuals about illness due to hookworm, vaccines, and participation in vaccine trials (Group 3).

The survey was first pilot tested on a group of 20 adults. After conducting this pilot test, modifications were made to improve the understanding of the tool by the general public. The survey was administered by specially trained interviewers at two distinct times: once immediately before viewing the educational video described above and then approximately two months later.

### Statistical Analysis

Data were tabulated and analyzed using the SPSS software program (version 15). Of the 45 questions on the questionnaire, only questions 2 through 40 were included in the analysis (the first question was for informational purposes whereas the final three concerned only those participants who had children). For questions from Groups 1 and 2, the frequencies of each answer were summarized. Responses to these questions were dichotomized such that each correct answer was assigned a value of “1” and each incorrect answer a value of “0”; a response of “don't know” was also assigned a value of “0” as it was considered a lack of knowledge. Subsequently, the mean of the responses was calculated for both the pre- and post-film administrations of the questionnaire. Additionally, composite scores for knowledge about hookworm and knowledge about the experimental hookworm vaccine were obtained by calculating the mean of responses to questions that fell into these broad categories. For the knowledge about hookworm composite score, responses to 14 similar questions were combined (#2–14, #26) whereas for knowledge about the hookworm vaccine 6 questions (#15, #16, #18–20, #30) were combined.

Questions in Group 3 were divided into two subgroups which were analyzed using different methods: for the first subgroup, answers were dichotomized such that an affirmative answer was assigned a value of “1” and a negative answer a value of “0” whereas for the second subgroup, the Likert scale consisting of five categories was used: −2 (strongly disagree), −1 (disagree), 0 (neither agree nor disagree), 1 (agree), and 2 (strongly agree). As for the dichotomous responses, means were calculated for each Likert scale question and compared pre- and post-film; to do this, the “strongly disagree” and “disagree” categories were combined and assigned a value of “0”, and the “strongly agree” and “agree” categories were assigned a value of “1”. As for the first two groups of questions, a composite score for the attitudes and feelings of study participants was created by combining individual responses to 7 different questions from the questionnaire (#21–23, #27–29, #31).

Student's paired t-test was used to compare pre-film and post-film means for both individual questions and the composite scores for Groups 1 and 2. The chi-square test was used to compare proportions for questions with several possible responses. For all tests, a *p* value less than 0.05 was considered significant.

## Results

Individuals were randomly selected from a list of participants who had been enrolled in a large epidemiological study and were invited to participate in the educational intervention. The initial sample consisted of 127 subjects; however, 55 of these did not undergo the second survey administration after viewing the educational video. The final study sample therefore consisted of 72 volunteers who were included in the data analysis. The average age of these participants was 30.1 years (range, 17 to 46 years), with 33 (45.8%) being female and 39 (54.2%) male.

### Knowledge about Hookworm and the Hookworm Vaccine

When assessed as a composite score, knowledge about hookworm improved significantly after viewing the informational video from a mean score of 0.68 before the video to 0.76 after the video (p<0.0001), demonstrating that significant understanding was acquired by the participants through the targeted educational intervention. When assessing knowledge about vaccines and the proposed clinical trials of the experimental *Na*-ASP-2 Hookworm Vaccine, a small improvement was also seen after viewing the video, with the mean of correct answers on the post-video questionnaire being significantly higher than the mean on the pre-video test (0.58 *vs*. 0.65, p = 0.03) (**Table 1**).

**Table 1:** Impact of the educational video on knowledge about hookworm and the experimental hookworm vaccine.

Composite Scores	Mean		Difference (95% CI)	*P* *
	Pre-video	Post-video		
Knowledge about hookworm^1^	0.68	0.76	0.09 (0.05–0.12)	0.0001
Knowledge about the hookworm vaccine^2^	0.58	0.65	0.07 (0.01–0.13)	0.03

*Paired Student's t-test comparing responses before and after viewing the educational video.

1 Composite of questions #2–14 and #26 from questionnaire.

2 Composite of questions #15, #16, #18–20 and #30 from questionnaire.

**Table 2** describes in more detail the specific knowledge acquired about hookworm after viewing the film. The illness, recognized by the popular name “amarelão” (“the big yellow”) by 88.9% of participants prior to viewing the film, was identified by the more scientific name “ancilostomídeo” by 91.7% of the study subjects after seeing the film (compared to only 79.2% before, p = 0.01). The mode of transmission of hookworm, which in Brazil is sometimes confused with other worm infections such as *A. lumbricoides* and *S. mansoni*, was already correctly identified as being through contact of skin with contaminated soil by 95.8% of subjects before the film, which increased to 100% after viewing the video (*p* = 0.08). However, the common misconception that hookworm infections are acquired through contact with contaminated water and unwashed fruit or vegetables, was abandoned by a quarter of the subjects participating in the research after viewing the educational film (88.9% pre-film *vs.* 63.9% post-film, p<0.001).

**Table 2:** Participants' responses to questions about hookworm infection, before and after the educational video intervention.

	Responses (%)				*P* *
	Pre-video		Post-video		
Question	Yes	No	Yes	No	
Have you heard about “amarelão^1^”?	88.9	11.1	100	0.0	0.004
Have you heard about “ancilostomídeo^2^”?	79.2	20.8	91.7	8.3	0.01
Can you have worms but not feel anything?	66.7	33.3	79.2	20.8	0.1
Is it possible that you are infected with hookworm/or the worms of “amarelão”?	70.8	29.2	84.7	15.3	0.03
Do you get infected with hookworm/worms of “amarelão” by walking barefoot or coming into contact with dirt?	95.8	4.2	100	0.0	0.08
Do you get infected with hookworm/worms of “amarelão” by eating unwashed fruits and vegetables or by drinking water?	88.9	11.1	63.9	36.1	<0.001
Does hookworm/“amarelão” cause anemia?	90.3	9.7	93.1	6.9	0.5
Does only hookworm/“amarelão” cause anemia?	38.9	61.1	33.3	66.7	0.4
Is hookworm a major health issue in your community?	84.7	15.3	91.7	8.3	0.2
Hookworm is not a serious illness because it can be easily treated.	72.2	27.8	45.8	54.2	<0.001
Hookworm can be treated with medications and once cured you can never get it again.	69.4	30.6	61.1	38.9	0.2
Hookworm can be eliminated from your community if hygienic practices are changed.	88.9	11.1	70.8	29.2	0.002
A fecal exam must be performed to know if you are infected with hookworm.	100	0.0	95.8	4.2	0.08
Infection with hookworm can cause serious long-term health problems.	91.7	8.3	84.7	15.3	0.1

*Paired Student's t-test comparing responses before and after viewing the educational video.

1 Brazilian Portuguese slang term for hookworm infection.

2 Brazilian Portuguese term for the hookworm parasite.

After the educational intervention, 79.2% of participants recognized that individuals can be infected with hookworm burt be asymptomatic, compared to 66.7% who held this view prior to seeing the film (p = 0.1). The acquisition of this knowledge may be crucial because health surveillance is intimately associated with the recognition of the gravity of the disease and the acknowledgement that it can be a “silent” illness. When asked about the negative health consequences associated with hookworm infection, even before viewing the educational film, 90.3% of participants correctly identified anemia as an important result of hookworm infection, compared to 93.1% after the film (p = 0.5).

Differences were found before and after the educational intervention when assessing the level of subjects' comprehension about the impact of illness due to hookworm in their community. Although even before viewing the video 84.7% of the population believed that hookworm was an important illness in their community, which increased slightly to 91.7% of participants afterward (p = 0.2), a significant change was seen in the more subtle question that asked whether participants thought that hookworm is not a serious illness because it can be easily treated: initially, 72.2% responded in the affirmative to this question whereas after viewing the educational film and hearing more about the long-term consequences of asymptomatic infection, only 45.8% held this viewpoint (p<0.001). Furthermore, after viewing the educational video more people understood that despite the existence of effective drug therapy for hookworm infection, there are major limitations with this treatment due to the probability of becoming re-infected, which in many cases can occur rapidly following treatment, although this increase in knowledge wasn't statistically significant (30.6% pre-video *vs.* 38.9% post-video, p = 0.2).

**Figure 1:**
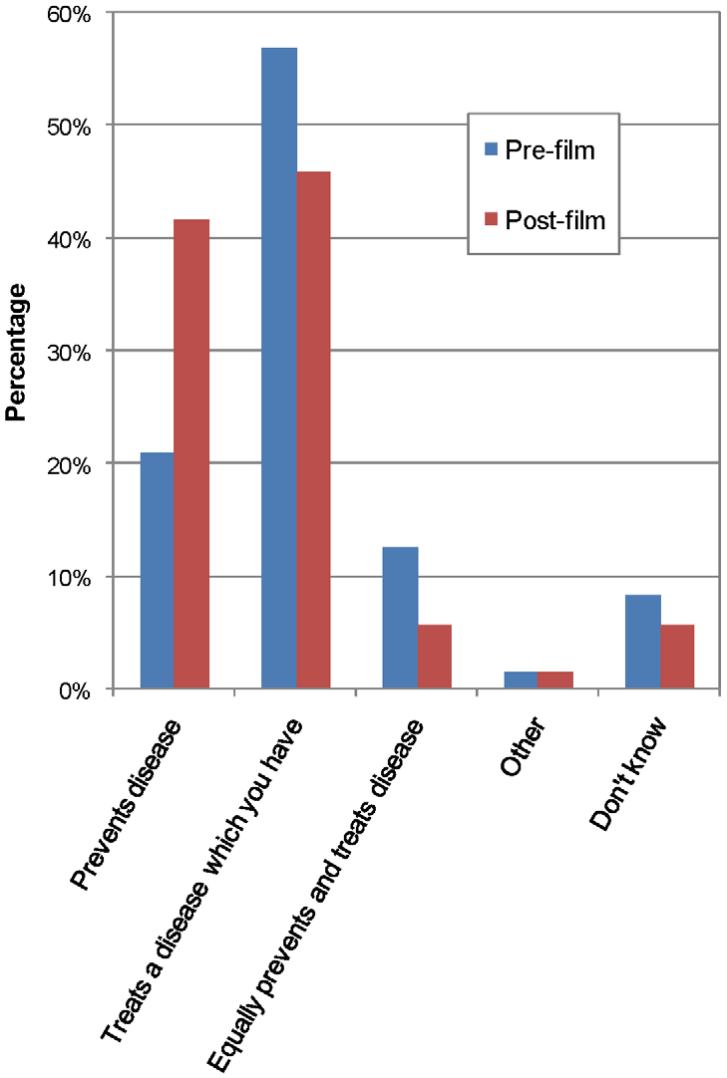
Knowledge of participants about the purpose of a vaccine, assessed before and after viewing the educational video.

When evaluating the participants' knowledge about vaccines in general and the upcoming hookworm vaccine trial in particular, improvements in understanding were acquired after viewing the educational film (**Table 3**). Regarding the purpose of vaccination, the participants' knowledge before and after the educational intervention is shown in **Figure 1**. Results from the pre-film survey demonstrate that a majority of participants (56.9%) believed that the purpose of a vaccine is to treat an established illness. Although following the film, this association was still made by almost half of responders (45.8%), a significant increase did occur in those associating a vaccine with illness prevention (41.7% post-video *vs.* 20.8% pre-video, p = 0.005).

**Table 3:** Participants' responses to questions about hookworm infection, before and after the educational video intervention.

	Responses (%)				*P* *
	Pre-video		Post-video		
Question	Yes	No	Yes	No	
Do you know that people are testing a vaccine against hookworm?	63.9	36.1	95.8	4.2	<0.001
Do you know what a vaccine does?	20.8	79.2	41.7	58.3	0.005
Will the expected results for a hookworm vaccine will be the same for everyone?	47.2	52.8	18.1	81.9	<0.001
If you are vaccinated, could you experience red and sore arms, headache, stomach ache, allergic reaction, death, or another reaction?	81.9	18.1	90.3	9.7	0.2
Will participants in the future hookworm vaccine study receive two different types of vaccine?	41.7	58.3	75.0	25.0	<0.001
Can only people who are sick participate in a vaccine study?	31.9	68.1	47.2	52.8	0.06

*Paired Student's titest comparing responses before and after viewing the educational video.

**Figure 2:**
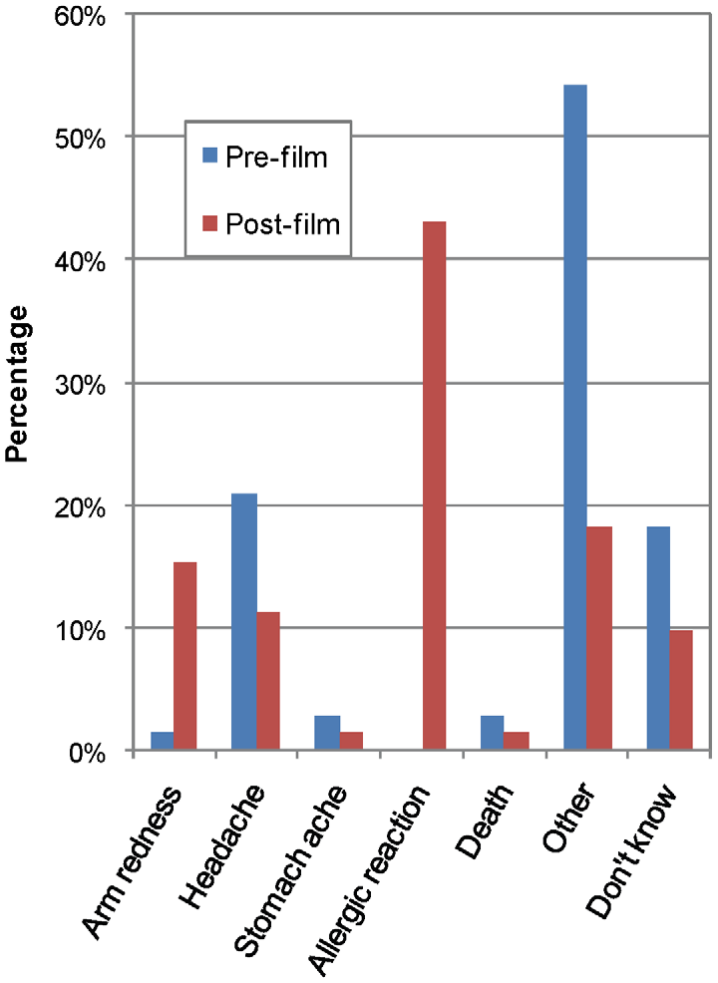
Knowledge of participants about the possible adverse effects associated with vaccination, assessed before and after the educational video.

To assess knowledge about the possible adverse effects caused by vaccination, participants were asked what they thought could happen if an experimental vaccine were administered to them. The potential side effects of vaccination that they chose are presented in **Figure 2**. Among the responses, the possibility of experiencing an allergic reaction upon being vaccinated, which was recognized by none of the interviewed participants prior to viewing the educational video, was cited frequently during the post-film test (43.1%), as was the possibility of experiencing arm redness (at the site of injection) following vaccination (15.3% post-video compared to 1.4% before, p = 0.003). Of note, the educational intervention was associated with a significant reduction in the proportion of individuals who answered “other” (18.1% post-film *vs.* 54.2% pre-film, p<0.001) and a non-significant reduction in those who chose “don't know” as their response to this question (9.7% post-film *vs.* 18.1% pre-film, p = 0.15).

### Attitudes and Perceptions

**Figure 3:**
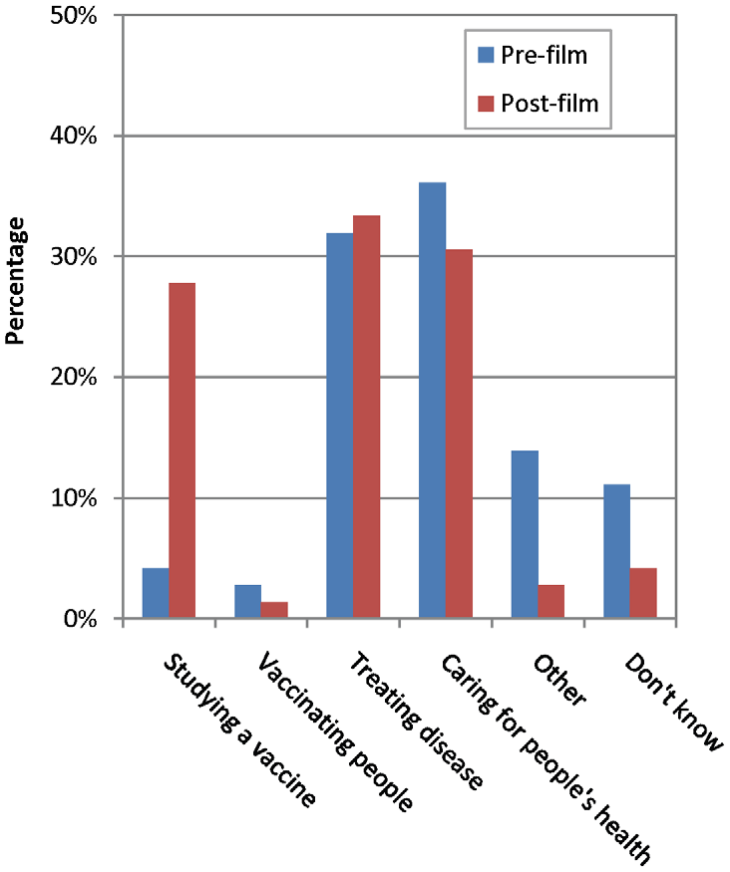
Perceptions of the participants about the role of the FIOCRUZ researchers in their community, assessed before and after the educational video.

The participants' perceptions about the nature of the work being done by the FIOCRUZ research team in the study area are shown in **Figure 3**. The level of comprehension regarding the work of the researchers underwent significant change following the presentation of the interventional video. Although the concept that the work of a researcher is to take care of people's health and treat disease remained the perception of 30.6% and 33.3% of the interviewed subjects, respectively, compared to 36.1% and 31.9% who listed these roles prior to viewing the film (p = 0.5 and 0.9, respectively), there was a significant increase in the proportion of participants who listed the function of the researcher as consisting of conducting studies on a new vaccine (27.8% post-film *vs.* 4.2% pre-film, p<0.001).

**Figure 4:**
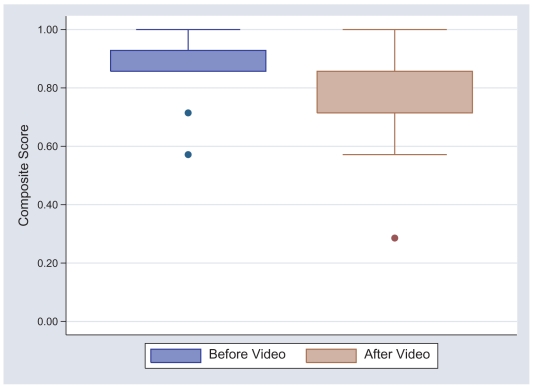
Comparison of responses to questions about the attitudes and perceptions towards the researchers and participation in vaccine clinical trials, both before and after the educational video. Unfavorable perceptions or attitudes were assigned a value of zero whereas favorable ones were assigned a value of one. Upper and lower limits of the boxes represent the interquartile ranges.

Regarding survey questions pertaining to the attitudes and feelings of the participants towards the researchers and participation in a hookworm vaccine trial, there was no significant change in the composite mean of responses before compared to after viewing the film (0.86 vs. 0.83, p = 0.07) (**Table 4**). Similarly, no significant difference was found between the attitudes and feelings expressed pre-film and post-film when those questions evaluated using the 5-point Likert scale were combined as shown in **Figure 4**. However, for both of these measures, the attitudes of the study participants were somewhat less favorable towards participating in future vaccine trials after viewing the informational video, even if these differences weren't statistically significant.

**Table 4:** Impact of the educational video on attitudes and perceptions about the research team and participation in future vaccine trials.

	Mean		Difference (95% CI)	*P* *
	Pre-video	Post-video		
Attitudes and feelings about the researchers and participating in a vaccine trial^1^	0.86	0.83	−0.03 (−0.003–0.06)	0.07

*Paired Student's t-test.

1 Combination of questions #21–23, #27–29 and #31 from questionnaire.

Even before the educational intervention took place, more than 95% of the participants interviewed already expressed a favorable attitude toward the planned vaccine study, displayed confidence in the work of the researchers and expressed interest in learning more about hookworm and the experimental hookworm vaccine (**Table 5**). In fact, 100% of respondents agreed that the researchers are doing good work in their community, a percentage that remained unchanged after viewing the film. After the educational intervention, although no significant changes were recorded in the attitudes and feelings regarding aspects of the hookworm vaccine project, there were reductions in the number of individuals who were interested in participating in a future vaccine trial (95.8% before compared to 88.9% after) and in those who said that their family would approve of their participation in a vaccine trial (86.1% before compared to 80.1% after).

**Table 5:** Participants' responses to questions about their attitudes and perceptions about aspects of the hookworm vaccine project, before and after viewing the educational video.

	Responses (%)					
	Pre-video			Post-video		
Question	Agree	Don't Agree	Don't Know	Agree	Don't Agree	Don't Know
Are you interested in participating in a hookworm vaccine study?	95.8	4.2	-	88.9	11.1	-
Would your family approve of your participation in a hookworm vaccine study?	86.1	13.9	-	80.1	19.9	-
Do you think that a hookworm vaccine study will help other people?	91.7	8.3	-	88.9	11.1	-
Are you interested in learning about hookworm and the vaccine?	97.2	2.8	-	100	0	-
Are the researchers doing good work in your community?	100	0	-	100	0	-
Do you trust the researchers working in your community?	97.2	2.8	-	95.8	4.0	-
Would you allow a vaccine to be tested on you if it is a new vaccine that has never been given to people before?	63.9	33.3	2.8	73.6	26.4	0.0
Does being a trial volunteer interfere with daily activities?	15.3	81.9	2.8	23.6	72.2	4.2
Are you scared of becoming sick if vaccinated?	29.2	70.8	0	51.4*	47.2	1.4
Could a hookworm vaccine help others?	86.1	8.3	5.6	93.1	1.4	5.6
Does being a vaccine trial volunteer improve your own health?	94.4	5.6	0	93.1	0	6.9
Will you receive attention and treatment for health problems if you participate in a vaccine trial?	95.8	2.8	1.4	93.1	0	6.9
Will you learn more about hookworm and health research if you participate in a vaccine study?	98.6	0	1.4	98.6	0	1.4
Will your family be proud of you if you participate in a vaccine trial?	86.1	9.7	4.2	72.3	19.4	8.3
Will you benefit from a vaccine trial?	93.0	4.2	2.8	84.7	11.1	4.2
Will participating in a vaccine trial be inconvenient for you?	23.6	70.8	5.6	27.8	66.7	5.6
Could the health researchers find a health problem you didn't know you had?	95.8	2.8	1.4	91.6	4.2	4.2
If you participate in a study, will information about you be kept secret?	59.7	31.9	8.2	65.3	23.6	11.1

*p<0.01

Closely related to these attitudes was the initial perception that participating in a vaccine study would result in focused attention on and treatment of health problems of study participants (95.8%), an aspect that certainly implies improvement of the health and the quality of life of each participating individual and indeed, even of others in the community (91.7%) (**Table 5** **)**.

Regarding the attitudes and feelings expressed by those surveyed toward the researchers and the hookworm vaccine project, it is important to note that the proportion of people expressing the opinion that being a volunteer in a research study may complicate daily activities or prove inconvenient increased slightly after viewing the video: 15.3% and 23.6% held these views prior to watching the film compared to 23.6% and 27.8% after (p = 0.2 and 0.07, respectively). Furthermore, the percentage of participants who expressed fear of becoming ill or experiencing an adverse event also increased significantly, from 29.2% to 51.4%, respectively (p<0.01).

## Discussion

Our study has demonstrated that despite conducting research in a rural, resource-limited population that has limited access to routine health care and education, specially-designed educational activities can significantly improve understanding and therefore the likelihood of obtaining truly *informed* consent for clinical research. The observed changes in the knowledge and perceptions of the research participants about hookworm infection and the experimental hookworm vaccine clearly demonstrate that the targeted educational intervention was successful in increasing understanding and that the subjects acquired knowledge pertinent to the planned research. Importantly, the increase in knowledge appeared to be sustained, as the post-video questionnaire was conducted two months following the viewing of the film and not immediately afterward.

The conceptual evolution following an educational intervention can be attributed to the non-cognitive educational methodology that was utilized. The video that was developed and evaluated as part of this study relied on the use of analogies – for example, comparing the production of a new vaccine to the making of a local sweet – to convey new scientific concepts related to hookworm and research. The analogy is a comparison based on similarities between the structures of two different fields of knowledge [27]. Reasoning through analogy is, therefore, a subjective internal process that is effectuated by the interaction between two mental fields. The results of the questionnaire demonstrate that this pedagogical methodology is effective in the population that was studied.

The educational approach chosen for this study differs from conventional educational methods, in that it considers the lifestyle of people, their ideas, beliefs and values, and the specific cultural context. This leads to enhanced self-esteem, increased community participation, thus promoting the values of citizenship [28]. As outlined by Rice and Candeias, the traditional educational model in which information is simply provided to and individual or community has only temporary effects in terms of behavior change [29]. When the educational stimulus ceases, so too are its effects. The main criticism of the traditional cognitive approach is that it does not take into consideration the psychosocial and cultural determinants of health behaviors [30].

In a previous study that we conducted in a rural area of Minas Gerais where schistosomiasis is endemic, different health education approaches were assessed regarding their effectiveness in increasing the knowledge of schoolchildren with respect to the transmission and prevention of this parasitic disease [31]. This study demonstrated that in a group of schoolchildren whose education was based upon a model using analogies or social representations, levels of knowledge about schistosomiasis increased significantly, compared to those in which a cognitive model based on the simple presentation of information, or a control group that received no specific information about the disease.

Regarding the specific changes in knowledge that were observed in the current study after the educational intervention, an increased appreciation of the illness caused by hookworm was observed, such that after watching the film, it was identified as an important affliction in the study area due to its endemnicity and its effect on people's lives. The understanding that hookworm infection is not a major health problem because it can be easily treated with anthelminthic medications was reduced following the film, serving as evidence of an improved understanding of the re-infection process wherein individuals are continuously at risk of infection despite anthelminthic treatment due to the environment in which they live.

In analyzing the attitudes and perceptions of the participants, it appears that there were no significant changes in the subjects' notions in relation to the researcher and the benefits of participating in clinical trials. Capturing the participants' perspective before and after the educational intervention, no change was observed. Initially, an overall favorable opinion toward the researchers and the project was observed, with a full 100% agreeing that the investigators are doing good work in their community. This good-will and trust towards the researchers remained unchanged after viewing the educational film, and it translated into a high level of interest in participating in future vaccine trials, although this willingness decreased slightly after viewing the film, perhaps as a result of an increased understanding of the risks involved, as discussed below.

According to the vast majority of the individuals who were studied, participating in a vaccine trial might not only benefit themselves but also bring benefit to others who are not participants, resulting in betterment of the community as a whole. Surprisingly, although some participants acknowledged that participating in a vaccine trial might interfere with their daily activities, the majority of those interviewed said that it would not be inconvenient to them. The time commitment involved, which for these individuals whose livelihoods depend on long hours of hard manual labor, could be significant but would apparently be outweighed by the perceived potential benefits of participation such as improvement of their own health, greater attention to treatment of health problems, and potential identification of health problems that are otherwise unrelated to the study or vaccination with an experimental product.

A broad increase in understanding of the work being conducted by the research team after viewing the educational film was observed among those surveyed, although the idea that the project's purpose is to treat illness and take care of people's health – instead of to conduct research – remained prevalent despite having viewed the video. Regarding this specific point, it is important to highlight that understanding the research process is a challenge for investigators who are involved in the preparation of communities in advance of conducting clinical trials and beginning the individual informed consent process, especially in resource-limited communities with low levels of literacy and limited access to routine health care [32].

Since the *Na*-ASP-2 Hookworm Vaccine is an experimental vaccine, individuals participating in the planned study of this investigational product could potentially experience adverse reactions of variable degrees of severity, although in the first clinical trial of this vaccine administered to healthy, hookworm-naïve volunteers living in the United States, observed reactions consisted mostly of mild to moderate intensity injection site reactions such as pain, swelling and erythema [33]. After viewing the film in which the potential risks of participating in the proposed vaccine trial were described, an increase in apprehension related to participation was observed. This should not necessarily be seen as a negative outcome of the educational video, as it may in fact reflect a superior understanding of the risks involved when participating as a volunteer in a clinical vaccine trial – something that should be welcomed. Even though the educational intervention may have resulted in fewer people who would be willing to participate (88.9% after watching the video compared to 95.8% before), if those individuals who remained interested in volunteering were better informed, the intervention was successful.

In contrast to an increased appreciation of the risks of participation in a vaccine trial, no significant changes were observed after the educational intervention in relation to the perceived benefits that might come from participating in the research. Among the perceived benefits of participating in a vaccine trial were improvements in health, treatment of illness, and a better quality of life.

As demonstrated by our findings, the educational intervention utilized in this study was not uniformly successful. In several instances, erroneous perceptions of the study participants – such as the belief that the purpose of a vaccine is to treat disease, or that the role of the research is to take care of the health problems of the population – persisted in a significant proportion of those interviewed even after viewing the educational video. Obviously, although these misconceptions were reduced by the use of the educational video, further work remains to better inform potential research participants of the nature of research and the purpose of interventions that might be tested in future research trials. As with any ongoing research project, obtaining informed consent from volunteers is an on-going process in which individuals are continuously engaged in educational activities.

In summary, not only is it important to assess the current level of understanding of potential vaccine trial participants prior to conducting clinical studies, it is also useful to design specially-tailored educational interventions to develop a more informed community that is able and willing to participate in such research. This process should be continuous, with frequent re-assessment of the understanding of individuals that results in revision of the educational materials. It is the ethical imperative of the investigators and research team to ensure that potential study participants have an adequate understanding of the research to be undertaken. In resource-limited areas such as our study site, this often requires more than simply reading the informed consent document, and may include targeted educational activities similar to the one tested at our study site.

## Supporting Information

Text S1 Survey of Community Knowledge, Attitudes, and Willingness to Participate in a Hookworm Vaccine Trial - Questionnaire Administered to Study Participants Before and After the Educational Video.
(0.10 MB PDF)

Video S1 Educational Video - Scene One (Introduction)
(3.36 MB MOV)

Video S2 Educational Video - Scene Two (What Is Hookworm?)
(8.58 MB MOV)

Video S3 Educational Video - Scene Three (How Do You Get Infected With Hookworm?)
(7.31 MB MOV)

Video S4 Educational Video - Scene Four (Community Advisory Boards)
(5.38 MB MOV)

Video S5 Educational Video - Scene Five (Who Can Participate In The Vaccine Trial?)
(9.01 MB MOV)

Video S6 Educational Video - Scene Six (The Hookworm Vaccine)
(4.20 MB MOV)

Video S7 Educational Video - Scene Seven (What Can Happen In The Vaccine Trial?)
(4.47 MB MOV)

Video S8 Educational Video - Scene Eight (The Hookworm Vaccine Trial)
(6.42 MB MOV)

Video S9 Educational Video - Scene Nine (The Final Product)
(4.06 MB MOV)
