# Supplementary material for: Health Education through Analogies: Preparation of a Community for Clinical Trials of a Vaccine against Hookworm in an Endemic Area of Brazil
Source: PLoS Negl Trop Dis. 2010 Jul 20;4(7):e749. doi: 10.1371/journal.pntd.0000749 (PMC2907418; doi:10.1371/journal.pntd.0000749)
Supplement: Text S1 — Survey of Community Knowledge, Attitudes, and Willingness to Participate in a Hookworm Vaccine Trial - Questionnaire Administered to Study Participants Before and After the Educational Video. (0.10 MB PDF) [file pntd.0000749.s001.pdf]

SURVEY OF COMMUNITY KNOWLEDGE, ATTITUDES, AND WILLINGNESS TO PARTICIPATE IN A HOOKWORM VACCINE TRIAL

Community: ____________ House: ______________ Identity: ______________

Name: ____

Date: ___ / _____ / ______ Interviewer: _______________________________

Knowledge

	No	Yes		
1. Have you participated in any meeting with researchers from the FIOCRUZ project?	1	2	How many? _____	☐
2. Have you heard of "yellow disease" ("amarelão")?	1	2		☐
3. Have you heard of "hookworm"?	1	2		☐

I will read some phrases to you and you will tell me if you think the statement is true, false, or if you don't know what they mean.

	True	False	Don't know	
4. Can you have worms but not feel anything?	1	2	8	☐
5. Is it possible that you are infected with hookworm/ or the worms of "amarelão"?	1	2	8	☐
6. Do you get infected with hookworm/worms of "amarelão" by walking barefoot or coming into contact with dirt?	1	2	8	☐
7. Do you get infected with hookworm/worms of "amarelão" by eating unwashed fruits and vegetables or by drinking water?	1	2	8	☐
8. Does hookworm/"amarelão" cause anemia?	1	2	8	☐
9. Does only hookworm/"amarelão" cause anemia?	1	2	8	☐
10. Is hookworm a major health issue in your community?	1	2	8	☐
11. Hookworm is not a serious illness because it can be easily treated.	1	2	8	☐
12. Hookworm can be treated with medications and once cured you can never get it again.	1	2	8	☐

		True	False	Don't know	
13.	Hookworm can be eliminated from your community if hygienic practices are changed.	1	2	8	☐
14.	A fecal exam must be performed to know if you are infected with hookworm.	1	2	8	☐

Vaccines/Researchers

		No	Yes	Don't know	
15.	Do you know that people are testing a vaccine against hookworm in your area?	1	2	8	☐

(The options for question #16 must not be read by the interviewer, so as not to influence the answers of the interviewee:)

16. Do you know what a vaccine does? ☐

1. It stops you from getting a disease
2. It treats a disease that you have
3. It both stops and treats diseases
4. I don't know what vaccines are for
5. Other
6. Don't know

		No	Yes	Don't know	
17.	If you receive the hookworm vaccine, will you be able to get infected with hookworm again?	1	2	8	☐
18.	Will the expected results for a hookworm vaccine will be the same for everyone?	1	2	8	☐

(The options for question #19 must not be read by the interviewer, so as not to influence the answers of the interviewee:)

19. If you are vaccinated, what could happen to you? ☐

1. Red and sore arms
2. Headache
3. Stomach ache
4. Allergy
5. Death
6. Other
7. Don't know

	**No**	**Yes**	**Don't know**	
20. Will participants in the future hookworm vaccine study receive two different types of vaccine?	1	2	8	☐
21. Are you interested in participating in a hookworm vaccine study?	1	2	8	☐
22. Would your family approve of your participation in a hookworm vaccine study?	1	2	8	☐
23. Do you think that a hookworm vaccine study will help other people?	1	2	8	☐

(The options for question #24 and #25 must not be read by the interviewer, so as not to influence the answers of the interviewee:)

24. What are the people from FIOCRUZ doing in your community? ☐

1. Studying a vaccine
2. Vaccinating people
3. Treating an illness
4. Taking care of people's health
5. Other
6. Research
7. Don't know

25. The researchers can bring some benefit to your community? What kind? ☐

1. Health
2. Financial
3. Quality of life
4. Gain knowledge
5. No benefit
6. Treatment
7. Clinic
8. Other
9. Don't know

Attitudes/Feelings

	**No**	**Yes**	**Don't know**	
26. Infection with hookworm can cause serious long-term health problems.	1	2	8	☐
27. Are you interested in learning more about hookworm and vaccines?	1	2	8	☐
28. Are the researchers doing good work in your community.	1	2	8	☐
29. Do you trust the researchers working in your community?	1	2	8	☐
30. Can only people who are sick participate in a vaccine study?	1	2	8	☐
31. Would you allow a vaccine to be tested on you if it is a new vaccine that has never been given to people before?	1	2	8	☐

I will read some phrases. Please tell me how you feel in relation to each statement.

		Strongly Disagree	Disagree	Neither agree nor disagree	Agree	Strongly Agree	
32.	Does being a trial volunteer interfere with daily activities?	1	2	3	4	5	☐
33.	Are you scared of becoming sick if vaccinated?	1	2	3	4	5	☐
34.	Could a hookworm vaccine help others?	1	2	3	4	5	☐
35.	Does being a vaccine trial volunteer improve your own health?	1	2	3	4	5	☐
36.	Will you receive attention and treatment for health problems if you participate in a vaccine trial?	1	2	3	4	5	☐
37.	Will you learn more about hookworm and health research if you participate in a vaccine study?	1	2	3	4	5	☐
38.	Will your family be proud of you if you participate in a vaccine trial?	1	2	3	4	5	☐
39.	Will you benefit from a vaccine trial?	1	2	3	4	5	☐
40.	Will participating in a vaccine trial be inconvenient for you?	1	2	3	4	5	☐
41.	Could the health researchers find a health problem you didn't know you had?	1	2	3	4	5	☐
42.	If you participate in a study, will information about you be kept secret?	1	2	3	4	5	☐

In the case of parents

If you have children up to 12 years of age, answer the following questions:

	No	Yes	Don't know		
43. If you are a mother/father, would you let your child participate in a health research study?	1	2	8	9	☐
44. Do you think that a study could interfere with the life of your child?	1	2	8	9	☐
45. Would your family approve of your child's participation in a study?	1	2	8	9	☐
